# Understanding Human Variation in Infectious Disease Susceptibility through Clinical and Cellular GWAS

**DOI:** 10.1371/journal.ppat.1003424

**Published:** 2013-08-01

**Authors:** Dennis C. Ko, Thomas J. Urban

**Affiliations:** 1 Department of Molecular Genetics and Microbiology, School of Medicine, Duke University, Durham, North Carolina, United States of America; 2 Department of Medicine, School of Medicine, Duke University, Durham, North Carolina, United States of and America; 3 Center for Human Genome Variation, School of Medicine, Duke University, Durham, North Carolina, United States of America; Duke University Medical Center, United States of America

Over the last ten years, advances in genotyping and high-throughput sequencing technologies have resulted in an explosion of genetic information. Whereas prior attempts at discovering human genetic differences affecting susceptibility to disease relied on genotyping one or a handful of candidate genetic variants, genome-wide association studies (GWAS) have now become a common means of searching for susceptibility genes in an unbiased way. These studies have highlighted the relevance of particular pathways in pathogenesis of infectious and autoimmune disease. Thus, GWAS of clinical phenotypes can alert host-pathogen researchers to unexpected links between their pathway of study and human disease. Complementary to this, cellular GWAS using pathogens as probes can reveal how genetic variation affects cellular processes important for disease pathogenesis.

## What Can GWAS Do for Cellular Microbiology? Identification of Genes and Pathways Important in Autoimmune and Infectious Disease Pathogenesis

In GWAS, controls and cases with a disease are genotyped at hundreds of thousands to millions of loci and the genotype frequencies are compared to identify alleles that may be protective or result in increased susceptibility [1]. Prior to the advent of GWAS, a handful of examples demonstrated that common genetic variation could have profound effects on infectious disease susceptibility. The sickle cell allele of hemoglobin protecting against malaria [2] and the CCR5 deletion allele protecting against HIV infection [3] are textbook examples. GWAS provide a way to systematically search for such genetic differences.

GWAS of autoimmune diseases have been particularly successful. For inflammatory bowel disease (IBD), a GWAS of 75,000 people revealed 163 loci that can account for ∼15% of the total disease variance of Crohn's disease [4]. The genomic regions implicated by these loci include genes showing a striking enrichment for immune-related gene ontology terms, including regulation of cytokine production and activation of lymphocyte signaling [4]. The causal variants, which nearby genes are affected, and how the affected genes alter pathophysiology are unknown for most of these loci. However, a few successful exceptions should spur researchers to mine this list of disease-relevant genetic differences. A frameshift mutation in *NOD2* that results in a truncated protein was identified as a Crohn's disease susceptibility allele [5], [6] prior to the GWAS era through linkage followed by candidate gene studies. NOD2 is a member of the NLR family of intracellular sensors that responds to both bacterial (muramyl dipeptide; MDP) and viral (ssRNA) patterns [7], [8]. Mice with the *NOD2* mutation had increased intestinal inflammation in the dextran sodium sulphate (DSS) model of colitis, and macrophages from the mice exhibited increased NF-κB signaling and IL-1β secretion in response to MDP [9]. The first reported GWAS hit for IBD was a non-synonymous mutation in an autophagy gene, *ATG16L1* [10]. Prior to this finding, autophagy had not been known to play a role in Crohn's disease susceptibility, and this discovery prompted further research in the interplay of autophagy, infection, and immunity. Recent cellular studies have linked these two susceptibility genes. NOD2 recruits ATG16L1 to the plasma membrane to cause autophagy of invasive bacteria [11], while a separate study showed that NOD2 activation by MDP enhances ATG16L1-mediated autophagy to increase antigen presentation in dendritic cells [12]. These studies of IBD demonstrate how careful and extensive follow-up of GWAS hits can be transformative to the understanding of pathophysiology. Researchers can determine whether their gene of interest has been implicated in GWAS by searching the NHGRI GWAS catalog [13] or the GWASdb website, which manually curates more hits and provides an easy-to-navigate browser [14].

Two successful examples from infectious disease GWAS further show how GWAS can inform our understanding of disease and even lead to changes in clinical practice. GWAS of leprosy have revealed eight loci affecting susceptibility [15], [16]. Overlap in leprosy and IBD-associated GWAS variants has clearly demonstrated the shared genetic underpinnings controlling susceptibility to infectious and autoimmune disease. Five of the eight loci associated with leprosy are also associated with Crohn's disease [4]. For example, an SNP upstream of *NOD2* protects against IBD but leads to increased risk of leprosy [4]. Thus, there are phenotypic trade-offs in genetic variation that may only be revealed under certain environmental situations.

GWAS of treatment response to hepatitis C virus (HCV) infection has prompted changes in clinical practice. Genetic variation at the IL28B locus (encoding interferon lambda-3) has been strongly associated with sustained virological response to pegylated interferon alpha plus ribavirin in patients with chronic HCV infection, with carriers of the beneficial genotype having 2- to 3-fold greater odds of eradicating the virus [17]. This effect appears to be particularly strong in patients with the G1 viral genotype [18]. Genotyping individuals for this variant has now become common in managing treatment [19]. While the clinical utility of IL28B genetic testing may begin to wane in the era of direct-acting anti-HCV drugs, the biological information gleaned from these studies will have lasting value, both in terms of recognizing the role of interferon lambdas in HCV infection, and perhaps as a novel route of therapeutic management [19].

Despite these successes, there are certainly limitations to GWAS [20], [21] and fewer examples of GWAS discoveries in infectious disease than for most common, chronic human diseases. Notably, sample sizes of infectious disease GWAS have been relatively modest compared to other GWAS. Lack of sufficient coverage of variation on genotyping platforms for African populations is also likely partially responsible. Selection of controls is also inherently difficult for infectious disease GWAS, especially for nosocomial and opportunistic infections for which information on pathogen exposure in uninfected individuals may be limited and patients have varied and multiple comorbidities that can lead to confounding. Perhaps most importantly, infectious disease GWAS have an additional source of genetic variation not present in GWAS of other human diseases—genetic variation in the pathogen. This is illustrated above with the recognition that the influence of IL28B genotype on HCV clearance is largely dependent on the viral genome. The host-pathogen arms race results in two moving targets and in some cases tremendous genetic heterogeneity. For example, HIV-1 is incredibly diverse, with thousands of genetically different viruses classified in a complex tree of types, subtypes, sub-subtypes, and recombinant forms [22]. Despite these challenges, loci associated with infectious disease typically have larger effect sizes compared to noninfectious disease GWAS and thus should be a priority [20]. Infectious disease GWAS will benefit from increased sample size, better coverage of variants in genotyping, and stratified analysis to minimize confounding due to variation in the pathogen.

## What Can Cellular Microbiology Do for GWAS? Cellular GWAS as a New Discovery Tool

**Figure 1:**
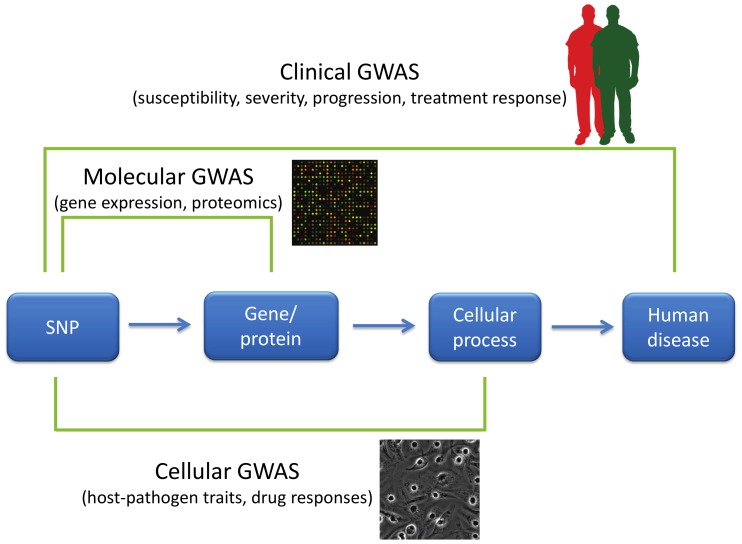
GWAS of varying phenotypic scales. GWAS have primarily been used to characterize disease-related characteristics in patient populations, but new approaches have expanded the phenotypes used in GWAS. “Clinical GWAS” search for associations between genetic differences (primarily in the form of SNPs) and human disease traits such as disease risk, severity of disease, disease progression, and response to treatment. “Molecular GWAS” search for associations between SNPs and molecular phenotypes such as levels of mRNAs, proteins, or metabolites. Finally, “cellular GWAS” connect SNPs to particular cellular processes. Phenotypic variation in these cellular processes can be examined by manipulation either pharmacologically or using pathogens.

While clinical GWAS have been successful at highlighting important pathways in disease pathogenesis, there is clearly a need for additional approaches directed at understanding how specific genetic variants affect disease. How can we more effectively move from lists of SNPs to greater biological insight? One approach is to complement the GWAS of organismal/clinical traits with GWAS of different phenotypic scales (Figure 1). For several years, molecular GWAS of gene expression have identified genes whose level of transcription is associated with nearby genetic variation (cis-eQTLs; expression quantitative trait loci [23], [24]). These resources are useful for GWAS researchers in trying to narrow down what genes are affected by functional genetic variants within a genomic region. Researchers can take advantage of online tools such as the eQTL Browser [25] to determine if there is human genetic variation near their gene of interest that may regulate levels of expression. The availability of cell lines with alternative alleles, as well as genome engineering approaches to introduce genetic differences into isogenic backgrounds [26], makes this an exercise that can lead to hypothesis-driven experiments to understand how human variants alter cell biology.

The flow of scientific inspiration can also proceed from cellular microbiology to GWAS. Inspired by the way cellular microbiology [27] has led to numerous key discoveries in basic cell biology, cellular GWAS approaches that utilize pathogens as probes can serve to connect cellular processes to human diseases. In a cellular GWAS, cells from hundreds of genotyped individuals are exposed to a stimulus and the varied responses serve as quantitative traits for GWAS. For example, in the platform developed by one of the authors ([28]; Hi-HOST: High throughput Human in vitrO Susceptibility Testing), cellular GWAS was carried out on the phenotype of pyroptosis [29], *Salmonella*-induced inflammatory cell death. Experimental follow-up of an eQTL near *APIP* (*apaf-1-interacting protein*) led to the discovery that APIP is an enzyme in the methionine salvage pathway and that this metabolic pathway regulates caspase-1 activation [30]. Genotyping data for this SNP in patients with the physiological criteria for sepsis suggested that the *APIP* allele that results in a more robust caspase-1 response in vitro reduced the odds of death [30]. These findings are now being examined further in an *APIP* mouse model and in patient populations with *Salmonella* infections. Pyroptosis is just one consequence of *Salmonella* infection, and by monitoring multiple cellular phenotypes, we are able to probe human variation in macropinocytosis (by measuring *Salmonella* invasion), endosomal biology (by measuring intracellular survival and replication), and numerous pro- and anti-inflammatory signaling pathways (by measuring the cytokine response).

Not only can cellular GWAS approaches help elucidate clinical GWAS of bacterial infections, but unexpected connections between cellular processes and noninfectious diseases may emerge from this approach. Increasing the number and types of stimuli will lead to a large catalog of cellular GWAS that have targeted various cellular processes. We have thus far focused on *Salmonella* and *Yersinia* phenotypes ([28], [30] and D. Ko, unpublished data), while a similar approach has been used for HIV [31]. Cellular GWAS have also been undertaken for several different drug responses [32], [33], and hits from a cellular GWAS of taxol sensitivity showed a statistically significant overlap with a clinical GWAS of peripheral neuropathy induced by this same drug in cancer patients [34]. Cellular GWAS can help to make sense of clinical GWAS associations by revealing what cellular processes may be involved and by providing an experimentally tractable system to allow for hypothesis testing.

## Perspective

For the last twenty years, cellular microbiology has provided amazing insights into the physiology of cells [27]. Cellular microbiology is now well poised to contribute to the field of human genetics. Pathogens have clearly been a driving force, and perhaps have even been the main selective pressure, during human evolution [35]. What better way to study functional consequences of common genetic variants that have undergone natural selection than with the agents that have driven that change? Discoveries await both cellular microbiologists and human geneticists, and the results should benefit our understanding of basic biology and susceptibility to disease.
